# OAF: a new member of the BRICHOS family

**DOI:** 10.1093/bioadv/vbac087

**Published:** 2022-11-24

**Authors:** Luis Sanchez-Pulido, Chris P Ponting

**Affiliations:** MRC Human Genetics Unit, Institute of Genetics and Cancer, University of Edinburgh, Edinburgh EH4 2XU, UK; MRC Human Genetics Unit, Institute of Genetics and Cancer, University of Edinburgh, Edinburgh EH4 2XU, UK

## Abstract

**Summary:**

The 10 known BRICHOS domain-containing proteins in humans have been linked to an unusually long list of pathologies, including cancer, obesity and two amyloid-like diseases. BRICHOS domains themselves have been described as intramolecular chaperones that act to prevent amyloid-like aggregation of their proteins' mature polypeptides. Using structural comparison of coevolution-based AlphaFold models and sequence conservation, we identified the Out at First (OAF) protein as a new member of the BRICHOS family in humans. OAF is an experimentally uncharacterized protein that has been proposed as a candidate biomarker for clinical management of coronavirus disease 2019 infections. Our analysis revealed how structural comparison of AlphaFold models can discover remote homology relationships and lead to a better understanding of BRICHOS domain molecular mechanism.

**Supplementary information:**

Supplementary data are available at *Bioinformatics Advances* online.

## 1 Introduction

The 10 human BRICHOS domain-containing proteins are divided among five subfamilies: proSP-C/SFTPC (one member), ITM/BRI (ITM2A, BRI2/ITM2B and BRI3/ITM2C), BRICD5 (one member), Gastrokines (GKN1, GKN2 and GKN3) and Tenomodulin/Chondromodulin (TNMD and CHM1/LECT1). For most, their precursors consist of three parts: an N-terminal transmembrane region, part of either a signal peptide for secretion or a signal-anchor for type II membrane proteins, followed by a long BRICHOS domain and a C-terminal shorter mature polypeptide generated by proteolytic cleavage. BRICHOS domain-containing proteins have been characterized as pre-pro-proteins, which once secreted, undergo proteolytic processing, usually by proprotein convertases, releasing their mature form (Chen *et al.*, 2022; Hedlund *et al.*, 2009; Knight *et al.*, 2013; Sanchez-Pulido *et al.*, 2002; Willander *et al.*, 2011). Furin-cleavage of the transmembrane protein BRI2, for example, yields a 23 amino acid polypeptide that may then be released from the precursor molecule. Two different mutations of its termination codon extend its reading frame, yielding neurotoxic polypeptides ABri and Adan 11 amino acids longer than normal (Vidal *et al.*, 1999, 2000). These extended polypeptides are deposited as amyloid fibrils causing neuropathologies called familial British and Danish dementias. Mutations in another member of the BRICHOS family, proSP-C (pro-Surfactant Protein C) also cause an amyloid-like disorder called interstitial lung disease, a heterogeneous group of respiratory pathologies that affect the normal function of the surfactant mono-layer that covers the alveoli and allows gas exchange in the lungs (Gustafsson *et al.*, 1999; Sáenz *et al.*, 2015).

Mature forms of some invertebrate members of the BRICHOS domain-containing family, such as arenicin, alvinellacin, nicomicin and capitellacin, have been characterized as antimicrobial polypeptides able to aggregate on lipid membranes and which exhibit detergent-like properties (Andrä *et al.*, 2008; Elliott *et al.*, 2020; Panteleev *et al.*, 2018; Safronova *et al.*, 2022; Shenkarev *et al.*, 2011; Tasiemski *et al.*, 2014).

The mature forms of BRICHOS proteins thus have a natural propensity to aggregate, including into amyloid-like fibrils. To prevent this, BRICHOS domains have evolved intramolecular chaperone functions, transporting their amyloidogenic cargo to appropriate cellular locations where their proteins' mature polypeptides are released following proteolytic cleavage (Chen *et al.*, 2022; Gharibyan *et al.*, 2022; Johansson *et al.*, 2006; Knight *et al.*, 2013; Landreh *et al.*, 2015; Sanchez-Pulido *et al.*, 2002; Willander *et al.*, 2011).

This anti-amyloidogenic function of BRICHOS domains has been explored as a potential therapeutic agent, seeking to prevent aggregation of the Aβ42 polypeptide in Alzheimer disease (Hermansson *et al.*, 2014; Manchanda *et al.*, 2022; Nerelius *et al.*, 2009; Poska *et al.*, 2016, 2020), the islet amyloid polypeptide (IAPP) in type 2 diabetes (Oskarsson *et al.*, 2018) or mutated NOTCH3 protein in CADASIL (cerebral autosomal dominant arteriopathy with subcortical infarcts and leukoencephalopathy) (Oliveira *et al.*, 2022).

To date only a single high-resolution structure of a BRICHOS domain has been determined (Willander *et al.*, 2012). More structural information, however, has recently been forthcoming from coevolution-based structure prediction algorithms, such as AlphaFold or trRosetta (Du *et al.*, 2021; Jumper *et al.*, 2021). Not only have these methods yielded a step-change in high-quality structure prediction, they have also substantially modified strategies used for computational protein analysis and remote homology identification (Monzon *et al.*, 2022; Sanchez-Pulido and Ponting, 2021).

Twenty years after the discovery of the BRICHOS domain (Sanchez-Pulido *et al.*, 2002), we decided to take advantage of these modified strategies and AlphaFold-predicted structures to undertake an in-depth exploration of the human BRICHOS domain family. Our investigation revealed a previously unknown human BRICHOS domain-containing protein, its putative proprotein convertase cleavage site and its associated mature 70 amino acid polypeptide, which we predict to have amyloidogenic properties.

## 2 Results and discussion

### 2.1 Structural comparison between proSP-C known structure and AlphaFold models of the BRICHOS family

**Fig. 1.**
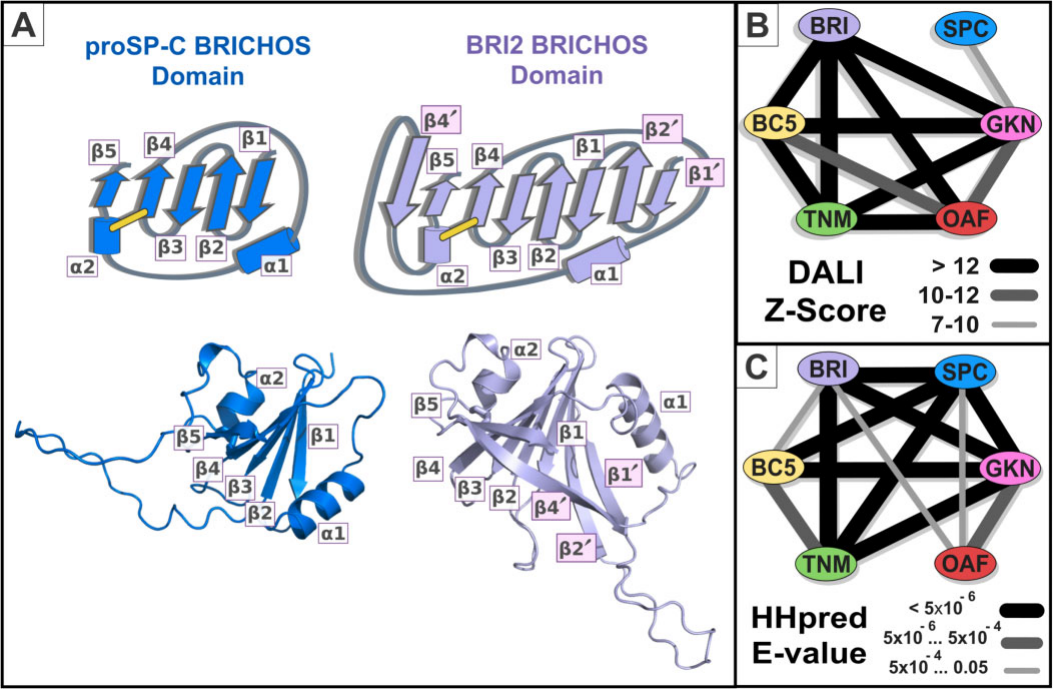
Structural and sequence analysis of BRICHOS. (**A**) Structural comparison of proSP-C and BRI2. Top: Secondary structure topology diagrams of proSP-C and BRI2. We adopt the nomenclature of secondary structural elements initially defined by Willander *et al.* (2012) for the proSP-C BRICHOS domain. Three additional β-strands can be found in the BRI2 AlphaFold model (labeled in pink: β-strands 1', 2' and 4'). Bottom: proSP-C and BRI2 BRICHOS domains AlphaFold models are shown, corresponding to positions 88–197 and 83–234, respectively. Cartoons of proSP-C and BRI2 were coloured in blue and light violet, respectively. (**B**) Z-Scores arising from Dali structural comparison searches against all human AlphaFold models, using as query a core BRICHOS domain AlphaFold structure for each human BRICHOS subfamily: ITM2B_HUMAN, residues 83–234 for ITMB/BRI2 (label BRI); PSPC_HUMAN residues 88–197 for SP-C; GKN2_HUMAN residues 20–151 for Gastrokine-2 (label GKN); BRID5_HUMAN residues 62–195 for BRICD5 (label BC5); TNMD_HUMAN residues 59–191 for Tenomodulin/Chondromodulin (label TNM); and OAF_HUMAN residues 27–172 for OAF. (**C**) The significance of profile-to-profile matches was evaluated in terms of an E-value, which estimates the number of observations of better sequence matches expected in a database by chance alone (Zimmermann *et al.*, 2018). The E-values correspond to HHpred searches against all Pfam profile database (including profiles independently generated for each human BRICHOS subfamily), using profiles of each human BRICHOS subfamily as query. For example, in an HHpred profile versus profile comparison search, the OAF profile matched the GKN (Gastrokine subfamily), BRI (ITM subfamily) and SPC (proSP-C subfamily) profiles with E-values 1.6 × 10^−4^, 0.008 and 0.015, respectively

The X-ray structure of proSP-C shows its BRICHOS domain to contain a central β-sheet composed of four consecutive anti-parallel β-strands followed by a fifth β-strand parallel to β-strand 4 (Fig. 1A) (Willander *et al.*, 2012). As expected, all AlphaFold BRICHOS domain models adopt the same topology, including two α-helices: one that is variably located, and another that is shorter and establishes a conserved disulphide bridge with β-strand 4 (Fig. 1A). Nevertheless, the proSP-C BRICHOS domain is atypical in two respects. First, it lacks three additional β-strands: two (β-strands 1' and 2') that continue the β-sheet at its N-terminus and a third that intervenes between β-strands 4 and 5 (Fig. 1A). Second, the SP-C mature polypeptide sequence is N-terminal to its BRICHOS domain, whereas it is C-terminal for all other human BRICHOS domain-containing proteins. In full-length proSP-C, this mature polypeptide is bound and stabilized by the groove (‘face A’) within the BRICHOS domain β-sheet (Willander *et al.*, 2012).

We started by inspecting all AlphaFold-predicted structures of human BRICHOS domain proteins (Tunyasuvunakool *et al.*, 2021), focusing first on the locations of their mature polypeptides. For the BRI2 model, this mature polypeptide is located within its face A binding groove (Supplementary Fig. S1A). Indeed, all-but-three mature polypeptides of human BRICHOS family members are located in this groove in models, consistent with the interacting surface identified experimentally for proSP-C (Willander *et al.*, 2012). For these three exceptions (Tenomodulin, Chondromodulin and proSP-C), their mature polypeptide lies outside of their models’ binding groove, as if it were a separate domain structure (Supplementary Fig. S1B). This major structural difference may reflect lower binding affinities between their BRICHOS domains and mature polypeptides, resulting in weaker evolutionary constraints and therefore different predicted tertiary structures. It is also plausible that AlphaFold is predicting only one of these proteins’ conformations, specifically either the mature polypeptide-bound or -unbound form.

### 2.2 Structural similarity searches against the AlphaFold human proteome

**Fig. 2.**
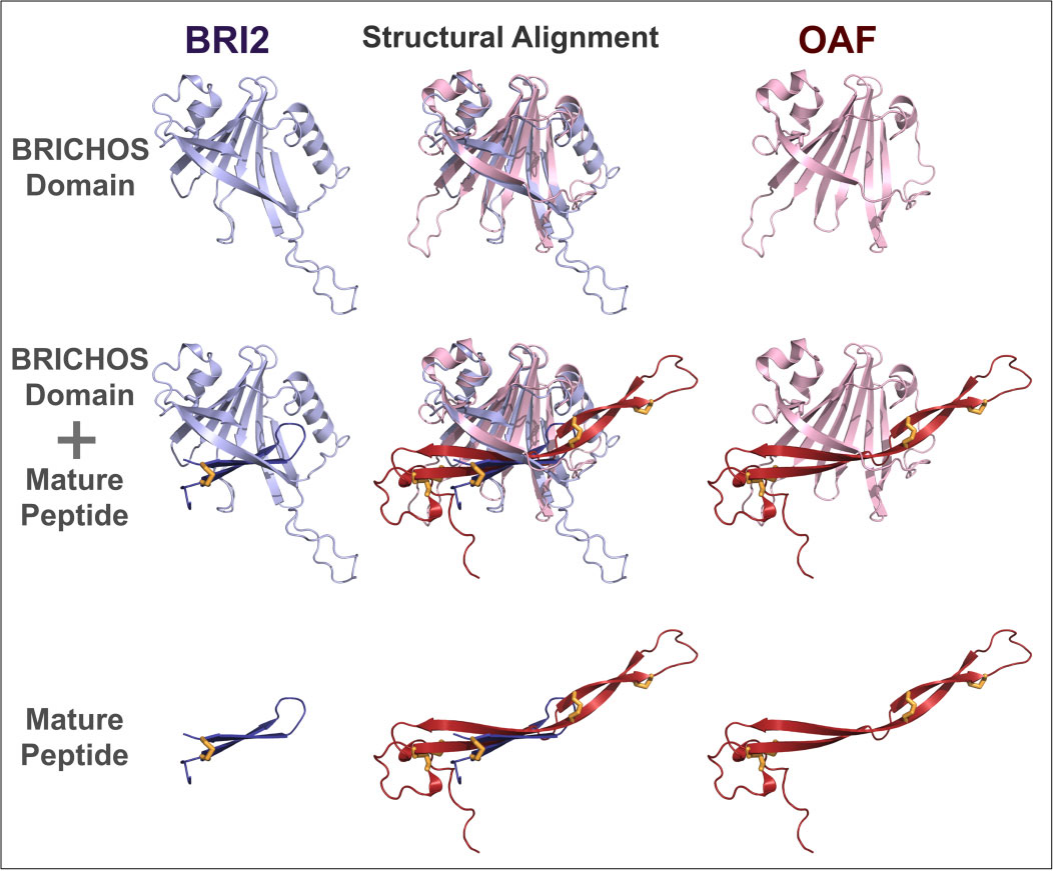
Structural superposition of BRI2 and OAF. Structural similarity of BRI2 and OAF AlphaFold model is shown. Top: BRI2 and OAF BRICHOS domains, corresponding to positions 84–233 and 29–172, respectively. Cartoons of BRI2 and OAF were coloured in violet and red, respectively. The BRI2 and OAF BRICHOS domains’ structural superposition (top row and middle column) was generated using Dali (Holm, 2022); other models in this figure are shown in this orientation. Middle: BRICHOS domains including their respective mature polypeptides. BRI2 and OAF mature polypeptide cartoons were coloured in a violet and dark red, respectively. Bottom: BRI2 and OAF mature polypeptides, corresponding to positions 244–266 and 204–273, respectively. Disulphide bridges in the mature polypeptides (one in BRI2 and four in OAF) are shown in yellow sticks. AlphaFold structural models were rendered using Pymol (http://www.pymol.org)

Structural comparison of BRICHOS AlphaFold models thus defined a conserved structural core for the BRICHOS domain, including β-strands β1’, β2’, β1–4, β4’ and β5, and the second α-helix (Supplementary Fig. S1). Subsequent structural searches with Dali (Holm, 2022) were undertaken against the AlphaFold human proteome (Tunyasuvunakool *et al.*, 2021), using human BRICHOS domain cores as query structures. Unexpectedly, these searches identified a new member of the human BRICHOS family, namely the OAF (Out at First) protein. Structural searches were convergent, identifying statistically significant structural similarity between OAF and different members of the BRICHOS family (Figs 1B and 2). No further human BRICHOS family domains were identified.

As structural similarity may result from either divergence from a common ancestor (i.e. homology) or else convergence, we next investigated amino acid sequence similarities between OAF and the BRICHOS domain sequences. For this, we performed a sequence conservation analysis using the HHpred profile-to-profile comparison tool (Zimmermann *et al.*, 2018). Statistically significant similarities were identified between OAF and BRICHOS domain sequences indicative of homology (Fig. 1C and Supplementary Fig. S2).

OAF shows five conserved features commonly found in BRICHOS family members (Knight *et al.*, 2013; Sanchez-Pulido *et al.*, 2002): (i) a predicted N-terminal transmembrane helix as part of a signal peptide facilitating secretion; (ii) a putative proprotein convertase (Furin or Furin-like) cleavage site, followed by, (iii) two anti-parallel β-strands likely covalently linked by disulphide bonds, whose nested arrangement of conserved cysteines within the predicted mature polypeptide is consistent with it adopting an extended anti-parallel hairpin structure (Fig. 2 and Supplementary Fig. S3); (iv) a predicted disulphide bridge between α-helix 2 and β-strand 4, whose conserved cysteines have been involved in a homopolymerization mechanism in reducing conditions, key for the ATP-independent chaperone function of these domains (Leppert *et al.*, 2022); and (v) a highly conserved aspartic acid (Asp74) located at the end of β-strand 2 (Supplementary Figs S2 and S3). This residue has been recently implicated in a pH-dependent regulatory mechanism of the BRICHOS domain's chaperone activity (Chen *et al.*, 2022). It is also expected to have a key functional relevance in BRICHOS domains because its mutation in proSP-C causes interstitial lung disease (Pobre-Piza *et al.*, 2022; Willander *et al.*, 2012).

Statistical significance of sequence and structural comparisons, and the presence in OAF of five features conserved among the BRICHOS family, are sufficient to infer that OAF is an 11th and previously unanticipated member of the human BRICHOS family.

### 2.3 The OAF family

*Oaf* was originally described in *Drosophila*, where its function was related to neuronal development and hatching (Bergstrom *et al.*, 1995). Phyletically, OAF is widely distributed in animals, including cnidarians, arthropods, annelids, molluscs, echinoderms and chordates, but it is absent from the nematode *Caenorhabditis elegans* (Pfam entry: PF14941) (Mistry *et al.*, 2021). Human OAF is poorly characterized experimentally and its physiological roles are unknown. It is ubiquitously expressed in human tissues, with high expression in brain (in particular in astrocytes), gastrointestinal tract, liver and respiratory system (The Human Protein Atlas; Uhlén *et al.*, 2015). It is also expressed highly in the eye's crystalline lens and is a candidate gene for Peters anomaly type 2 (involving corneal opacity) and ectopia lentis (dislocation or displacement of the lens) (David *et al.*, 2018). Oaf gene knockout mice exhibit abnormal eye phenotypes (International Mouse Phenotyping Consortium; http://www.mousephenotype.org/data/genes/MGI:94852).

Down-regulation of OAF in kidney has been associated to defects in tubular re-uptake of albumin (Teumer *et al.*, 2019). In agreement with this, knockdown of *Oaf* in *Drosophila* nephrocytes reduces albumin endocytosis (Teumer *et al.*, 2019).

A de novo heterozygous missense mutation (T171I) has been recently identified in OAF, and described as a putative cause of a musculoskeletal and neurological developmental disorder (Kaplanis *et al.*, 2020). Human OAF residue T171 is well conserved across vertebrates and is located at the end of its BRICHOS domain (Supplementary Figs S2 and S3). Its mutation to isoleucine is predicted by PolyPhen2 to be probably damaging (score: 0.992; sensitivity: 0.70; specificity: 0.97) (Adzhubei *et al.*, 2010).

OAF’s predicted mature polypeptide is longer than for other BRICHOS family members, containing 70 residues (residues 204–273), three-times longer than the BRI2 mature peptide (23 amino acids), for example. It is likely to be highly stable, owing to its four conserved disulphide bridges (Fig. 2 and Supplementary Fig. S3) and its sequence is highly conserved (48% identity between human OAF and *Drosophila melanogaster* Oaf), indicative of functional conservation.

Other mature polypeptides of the BRICHOS family form amyloid-like structures (Chen *et al.*, 2022; Hedlund *et al.*, 2009; Knight *et al.*, 2013; Willander *et al.*, 2011). To investigate this for OAF, we applied a machine learning approach, AMYPred-FRL, to its wild-type sequence (Charoenkwan *et al.*, 2022). This predicted OAF to have the highest probability (97%) of forming amyloid-like structures among all BRICHOS domain mature polypeptides (Supplementary Fig. S4A). This is consistent with the OAF BRICHOS domain having an intramolecular chaperone function that hinders aggregation of its mature polypeptide.

OAF protein is a candidate biomarker for progression and clinical management of pulmonary tuberculosis and coronavirus disease 2019 (COVID-19)-associated pneumonia, owing to its 1.3-fold expression levels increase in untreated tuberculosis patients versus healthy controls (Lu *et al.*, 2022), and its approximately 1.6- to 2.2-fold greater abundance in sera or plasma from critical versus non-critical cases of COVID-19 (Calvet *et al.*, 2022; Di *et al.*, 2020). We note that SARS-CoV-2 infection complications in lungs and brain, such as acute respiratory distress syndrome and acute neurological disorder, have been proposed to be amyloid-related pathologies (Ziff *et al.*, 2022; Sinha and Thakur, 2021) and, furthermore, impaired amyloid processing has been implicated in patients with COVID-19-associated neurological syndromes (Ziff *et al.*, 2022). Experimental investigation of whether OAF mature polypeptide forms amyloid structures (Supplementary Fig. S4B) or whether the OAF BRICHOS domain is anti-amyloidogenic in COVID-19 and other disease contexts is thus justified.

## 3 Conclusions

Our analysis showed that structural comparison of AlphaFold models can reveal remote homology relationships and details of polypeptide binding surfaces leading to a better understanding of molecular mechanism. More specifically, the identification of a BRICHOS domain in OAF will aid the design of future experiments that investigate its physiological function and potential role in the aetiology of amyloid-like diseases.

## Supplementary Material

vbac087_Supplementary_Data
