# Supplementary material for: OAF: a new member of the BRICHOS family
Source: Bioinform Adv. 2022 Nov 24;2(1):vbac087. doi: 10.1093/bioadv/vbac087 (PMC9714404; doi:10.1093/bioadv/vbac087)
Supplement: vbac087_Supplementary_Data [file vbac087_supplementary_data.zip › SanchezPulidoOAFSupplemental.pdf]

OAF, a new member of the BRICHOS family.

Supplemental Figure S1. BRICHOS family.

A) Structural superposition of proSP-C and BRI2. Top: ProSP-C and BRI2 BRICHOS domains, corresponding to positions 88-197 and 83-234, respectively. Cartoons of proSP-C and BRI2 were colored in blue and light violet, respectively. The proSP-C and BRI2 BRICHOS domains' structural superposition (top row middle column) was generated using Dali (Holm, 2022); other models in this figure are shown this orientation. Bottom: The red oval over proSP-C Face A indicates the polypeptide interacting surface identified using HDX-MS (Hydrogen Deuterium Exchange Mass Spectrometry) (Willander *et al.*, 2012). BRI2 mature polypeptide cartoon is coloured in dark blue (corresponding to positions 244-246). AlphaFold structural models were rendered using Pymol (http://www.pymol.org).

B) Structural superposition of BRI2 (left) and Tenomodulin (TNMD; right) AlphaFold structural models. Cartoons of BRI2 and Tenomodulin BRICHOS domains were colored in light violet and green, respectively. Middle: BRI2 and Tenomodulin structural superposition was generated using Dali (Holm, 2022); other models are shown in this orientation. The BRI2 mature polypeptide cartoon is coloured in dark blue (corresponding to positions 244-246), and the C-terminal region of Tenomodulin is coloured in black. The structural superposition of diverse BRICHOS domains show a high variability in the position of the α-helix 1 (see proSP-C/BRI2 or BRI2/TNMD BRICHOS domains superposition in A and B, respectively), therefore we decided not to include α-helix 1 in the BRICHOS domain structural core definition.

Supplemental Figure S2. Multiple sequence alignments of the BRICHOS domain.

This alignment was generated using a combination of profile-to-profile comparisons (Zimmermann *et al.*, 2018) and sequence alignments derived from structural superpositions of AlphaFold models (Holm, 2022). The amino acid colouring scheme indicates the average BLOSUM62 score (correlated to amino acid conservation) in each alignment column: red (greater than 3), violet (between 3 and 1) and light yellow (between 1 and 0.3).

The evolutionarily conserved disulphide bridge inside the BRICHOS domains (between α-helix 2 and β-strand 4) is linked with a yellow line. The OAF Asp74 is labelled (located at the end of β-strand 2, corresponding to proSP-C Asp105 and BRI2 Asp148). The human OAF Thr171Ile mutation is labelled. Sequences are named according to their UniProt identifier. For species abbreviations see Figure S3 legend.

Supplemental Figure S3. Representative full-length multiple sequence alignments of the OAF family.

Three coloured rectangles indicate conserved regions in the OAF family: a N-terminal transmembrane region (in green), a predicted BRICHOS domain (in red) and the C-terminal cysteine-rich putative mature polypeptide (in blue). Our evidence to propose a possible cleavage site of proprotein convertases (furin or furin-like proteases) in OAF was based on i) conservation and ii) location of the arginine in position 203 (in human OAF). The putative human cleavage site does not fit the canonical furin motif (RXXR). Nevertheless, the OAF family alignment shows that some family members contain a motif more consistent with this canonical furin site (for example: A0A6P4ZMP4_BRABE). This is the location where the furin cleavage site lies in other BRICHOS family members. Cysteines predicted in the human OAF AlphaFold model to contribute to disulphide bridges (one within the BRICHOS domain and four in the putative mature peptide) are linked with yellow lines. An additional disulphide bridge, putatively present in other members of the OAF family (for example, in *Drosophila*), is shown bellow the alignment.

This OAF family multiple sequence alignment was generated with the program T-Coffee (https://tcoffee.crg.eu/) using default parameters, slightly refined manually and visualized with the Belvu program (https://sonnhammer.sbc.su.se/Belvu.html). The amino acid colouring scheme indicates the average BLOSUM62 score (correlated to amino acid conservation) in each alignment column: red (greater than 3.5), violet (between 3.5 and 1.5) and light yellow (between 1.5 and 0.5).

The putative OAF signal peptide and protease cleavage sites, are labelled, as are OAF Asp74 and the Thr171Ile mutation. Sequences are named according to their UniProt identifier. Species abbreviations: 9CRUS, *Argulus foliaceus*; 9EUPU, *Arion vulgaris*; ACTTE, *Actinia tenebrosa*; ANODA, *Anopheles darlingi*; BRABE, *Branchiostoma belcheri;* BUGNE, *Bugula neritina*; DANRE, *Danio rerio*; DROME, *Drosophila melanogaster*; HELRO, *Helobdella robusta*; HUMAN, *Homo sapiens*; OCTVU, *Octopus vulgaris*; PENVA, *Penaeus vannamei*; STEMI, *Stegodyphus mimosarum.*

Supplemental Figure S4. Amyloid tendencies in BRICHOS domain-containing proteins.

A) AMYPred-FRL, a machine learning based tool for identification of amyloid proteins, predicts all mature polypeptides of human BRICHOS proteins within an Amyloid (AMY) class with high amyloid tendency probabilities (Prob) (Charoenkwan *et al.*, 2022). Only 1.1% of *Saccharomyces cerevisiae* proteins are associated with probabilities exceeding 95% (Charoenkwan *et al.*, 2022).

B) Schematic diagram of pre-pro-protein maturation that has been experimentally characterised for BRI2 and is hypothesised for OAF. Right: Amyloid pathologies potentially linked to their mature polypeptide forms.
