# Supplementary figures and images for: OAF: a new member of the BRICHOS family
Source: Bioinform Adv. 2022 Nov 24;2(1):vbac087. doi: 10.1093/bioadv/vbac087 (PMC9714404; doi:10.1093/bioadv/vbac087)

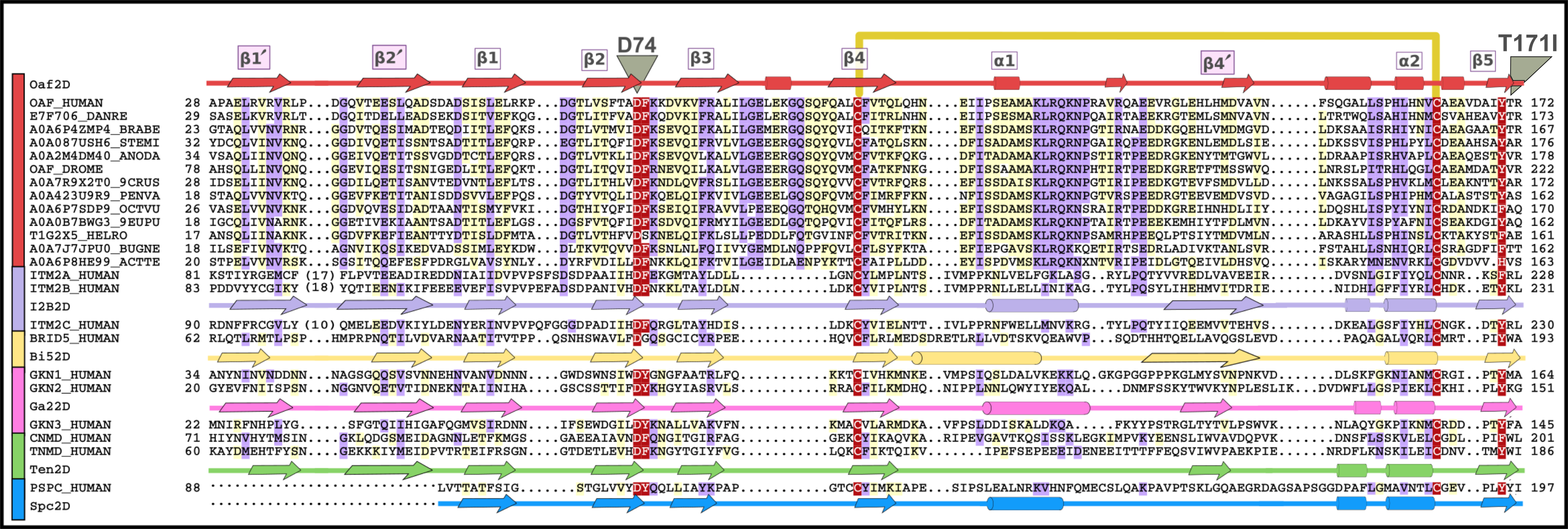

Supplement: vbac087_Supplementary_Data [file vbac087_supplementary_data.zip › S2.png]

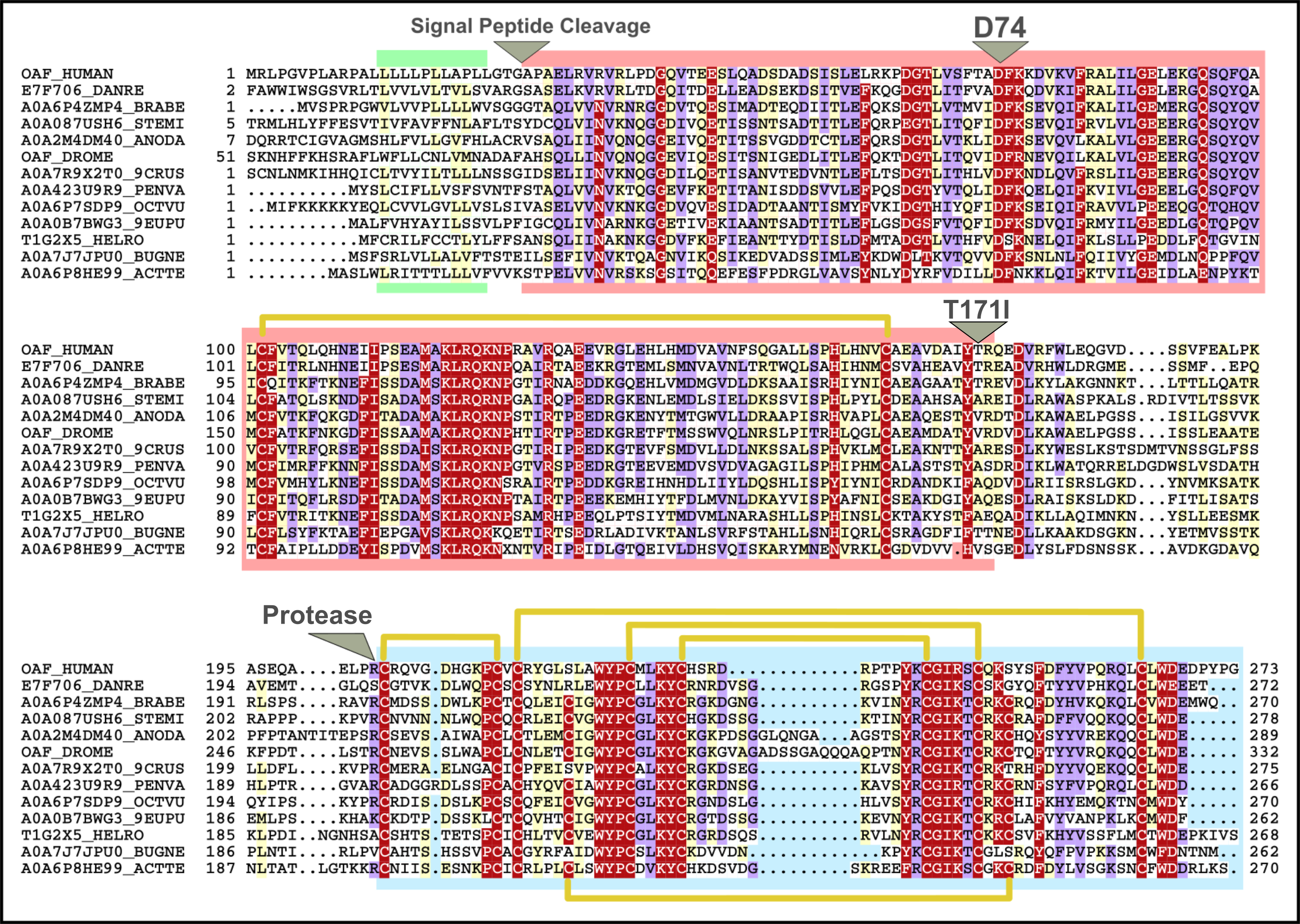

Supplement: vbac087_Supplementary_Data [file vbac087_supplementary_data.zip › S3.png]

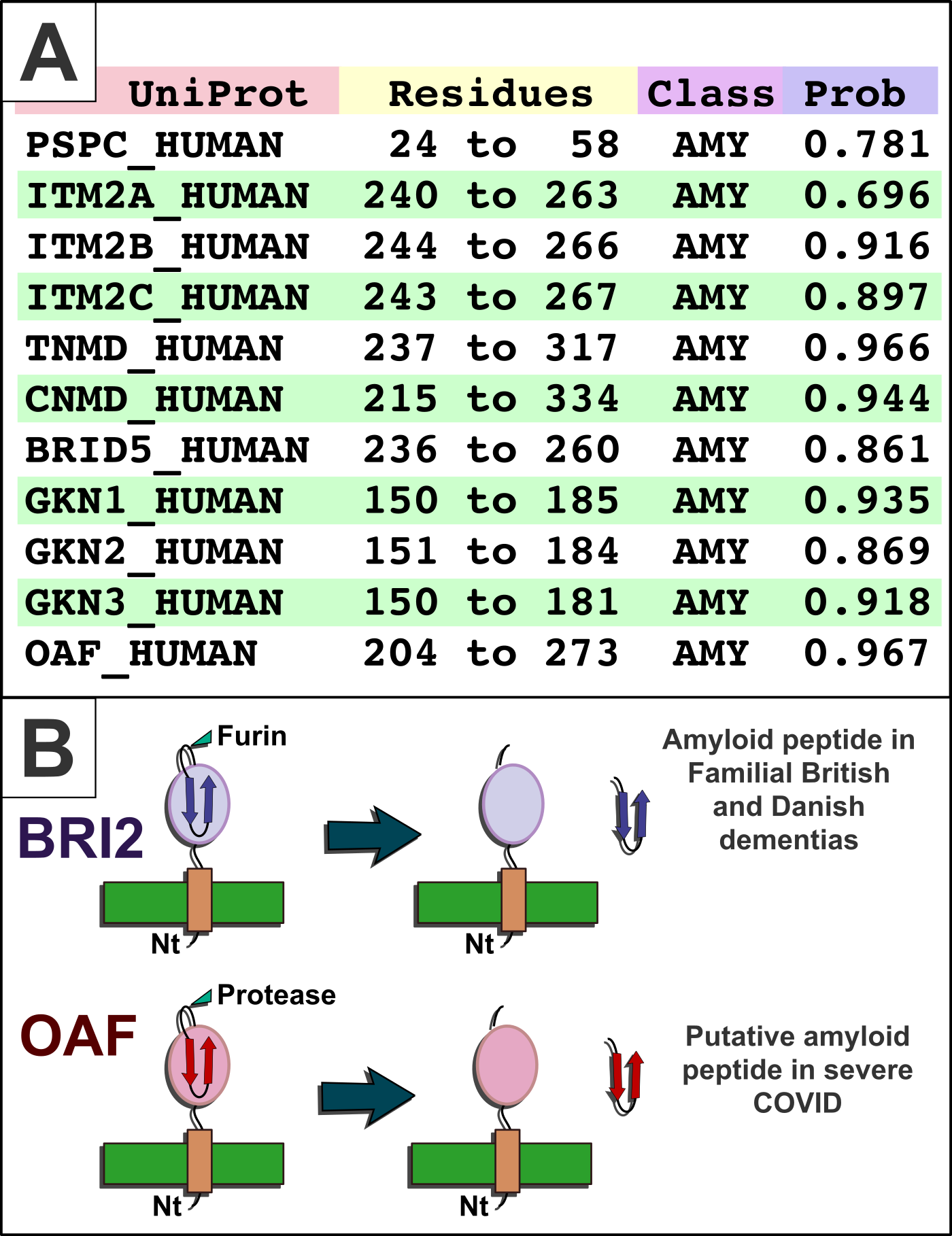

Supplement: vbac087_Supplementary_Data [file vbac087_supplementary_data.zip › S4.png]

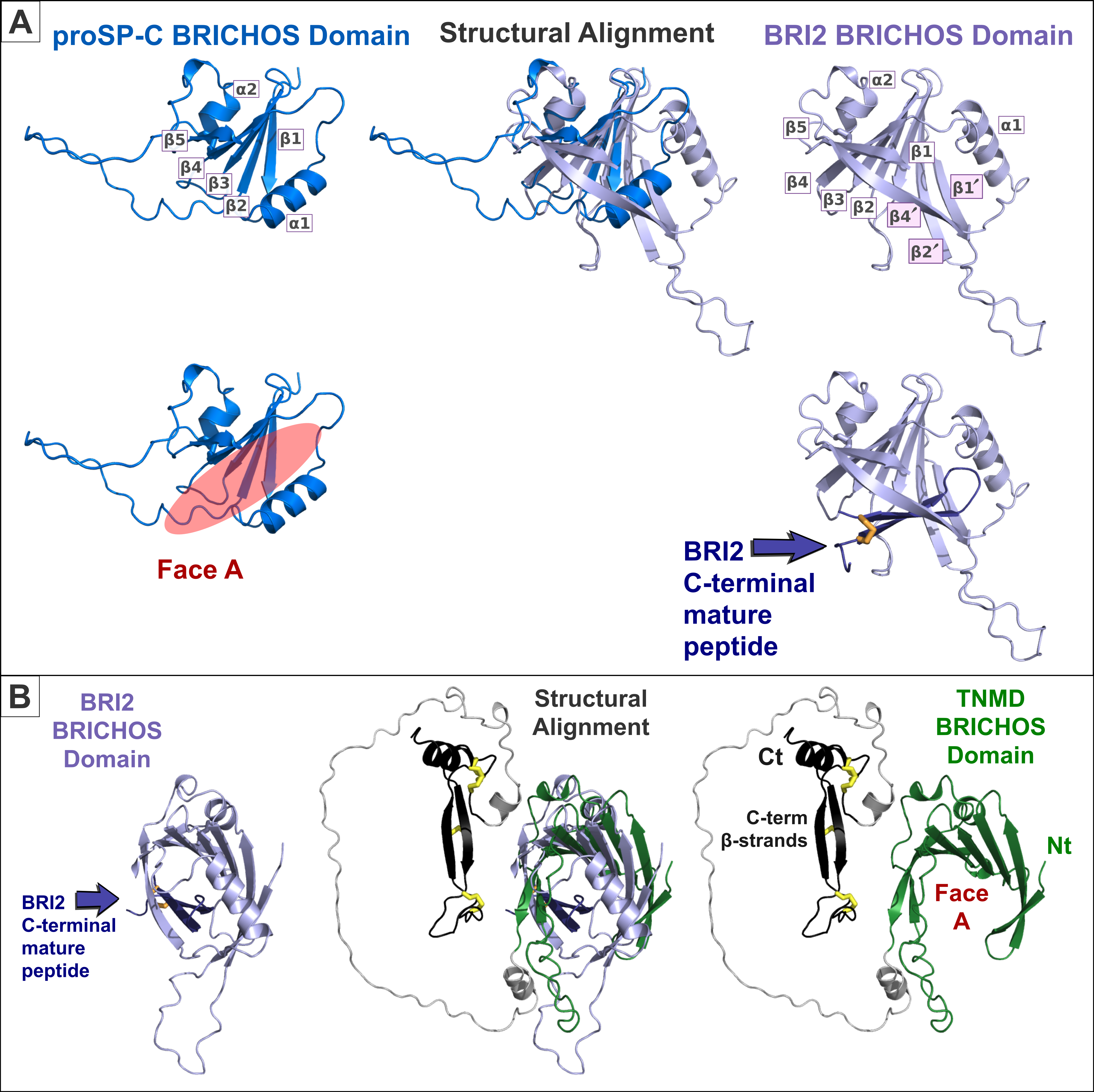

Supplement: vbac087_Supplementary_Data [file vbac087_supplementary_data.zip › S1.png]
